# Association of the Sirtuin and Mitochondrial Uncoupling Protein Genes with Carotid Plaque

**DOI:** 10.1371/journal.pone.0027157

**Published:** 2011-11-07

**Authors:** Chuanhui Dong, David Della-Morte, Liyong Wang, Digna Cabral, Ashley Beecham, Mark S. McClendon, Corneliu C. Luca, Susan H. Blanton, Ralph L. Sacco, Tatjana Rundek

**Affiliations:** 1 Department of Neurology, Miller School of Medicine, University of Miami, Miami, Florida, United States of America; 2 John T. McDonald Department of Human Genetics, John P. Hussman Institute for Human Genomics Miller School of Medicine, University of Miami, Miami, Florida, United States of America; 3 Department of Epidemiology and Public Health, Miller School of Medicine, University of Miami, Miami, Florida, United States of America; 4 Department of Laboratory Medicine and Advanced Biotechnologies, IRCCS San Raffaele Pisana, Rome, Italy; Innsbruck Medical University, Austria

## Abstract

**Objective:**

Sirtuins (SIRTs) and mitochondrial uncoupling proteins (UCPs) have been implicated in cardiovascular diseases through the control of reactive oxygen species production. This study sought to investigate the association between genetic variants in the SIRT and UCP genes and carotid plaque.

**Methods:**

In a group of 1018 stroke-free subjects from the Northern Manhattan Study with high-definition carotid ultrasonography and genotyping, we investigated the associations of 85 single nucleotide polymorphisms (SNPs) in the 11 SIRT and UCP genes with the presence and number of carotid plaques, and evaluated interactions of SNPs with sex, smoking, diabetes and hypertension as well as interactions between SNPs significantly associated with carotid plaque.

**Results:**

Overall, 60% of subjects had carotid plaques. After adjustment for demographic and vascular risk factors, T-carriers of the SIRT6 SNP rs107251 had an increased risk for carotid plaque (odds ratio, OR = 1.71, 95% CI = 1.23–2.37, Bonferroni-corrected p = 0.03) and for a number of plaques (rate ratio, RR = 1.31, 1.18–1.45, Bonferroni-corrected p = 1.4×10^−5^), whereas T-carriers of the UCP5 SNP rs5977238 had an decreased risk for carotid plaque (OR = 0.49, 95% CI = 0.32–0.74, Bonferroni-corrected p = 0.02) and plaque number (RR = 0.64, 95% CI = 0.52–0.78, Bonferroni-corrected p = 4.9×10^−4^). Some interactions with a nominal p≤0.01 were found between sex and SNPs in the UCP1 and UCP3 gene; between smoking, diabetes, hypertension and SNPs in UCP5 and SIRT5; and between SNPs in the UCP5 gene and the UCP1, SIRT1, SIRT3, SIRT5, and SIRT6 genes in association with plaque phenotypes.

**Conclusion:**

We observed significant associations between genetic variants in the SIRT6 and UCP5 genes and atherosclerotic plaque. We also found potential effect modifications by sex, smoking and vascular risk factors of the SIRT/UCP genes in the associations with atherosclerotic plaque. Further studies are needed to validate our observations.

## Introduction

Atherosclerosis is a complex disorder and underlying cause of ischemic strokes and cardiovascular diseases (CVD) [1]. Presence of carotid plaque has been widely used to assess the risk of future clinical atherosclerotic disease. Atherosclerotic plaque reflects biologically distinct atherosclerotic phenotype [1]. The heritability of carotid plaque is 23–50%, indicating an important role of genetic contribution to atherosclerosis [2], [3]. Genes controlling the oxidative stress, balance between production and removal of reactive oxygen species (ROS), are strongly implicated in mechanisms of atherosclerosis, stroke and cardiovascular disease (CVD) [4]. Oxidative stress plays a major role in age-dependent atherosclerosis by the enhancement of endothelial dysfunction and reduction of nitric oxide (NO) bioactivity, determining vascular aging independently of other traditional vascular risk factors [5].

Sirtuins (SIRTs) are a family of nicotinamide adenine dinucleotide (NAD^+^)–dependent deacetylases involved in chromatin remodeling, cellular metabolism and lifespan regulation. [6] Mitochondrial Uncoupling Proteins (UCPs) are a family of inner mitochondrial membrane proteins capable of driving the ATP synthase pathway via regulation of the proton electrochemical gradient [7]. SIRTs and UCPs may modify the oxidative stress and therefore affect the risk of atherosclerosis [6], [8], [9]. Moreover, the up or down regulation and the enzymatic activity of SIRT/UCP proteins have been related to the degree of tolerance to brain ischemia [10].

Associations of SIRTs and UCPs with the traditional vascular risk factors (RF) have been previously reported. Variants of the SIRT1, SIRT2, SIRT6, UCP1, UCP2, and UCP3 genes have been related to diabetes [11], obesity [12], [13], serum high-density lipoprotein cholesterol (HDL) [14], and inflammation [15]. However, few studies have examined the direct association between these genes and carotid atherosclerotic plaque, a subclinical marker of vascular disease [16].

We sought to examine the associations between variance of the 6 SIRT and 5 UCP genes and the presence and number of carotid plaque in a stroke-free population from a population based cohort. Based on our previous observation that SIRT1/UCP2 pathways protect against cerebral ischemia with a synergistic effects in maintaining oxidative balance and ATP production, resulting in an increase in cellular survival and modification in response to different stimuli, such as oxidative stress [10], we hypothesized that variants in sirtuin and UCP genes may have functional significance in the pathophysiology of subclinical vascular disease.

## Materials and Methods

### Subjects

This study consisted of a sample of 1018 stroke-free participants from the Northern Manhattan Study (NOMAS) who had carotid ultrasound performed and a genome-wide association (GWAS) data available. We have reported the detailed ascertainment scheme of the NOMAS previously [17]. Briefly, NOMAS participants were eligible if they had never been diagnosed with a stroke, were at least 40 years of age, and resided for at least 3 months in a household with a telephone in northern Manhattan. At enrollment, demographic characteristics and RF were collected through standardized questionnaires and laboratory tests. Hypertension was defined as a systolic blood pressure ≥140 mmHg, or diastolic blood pressure ≥90 mmHg, or a history of hypertension and anti-hypertension treatment; diabetes mellitus was defined as fasting blood glucose ≥126 mg/dl, or use of insulin or hypoglycemic medications; hypercholesterolemia was defined as total cholesterol ≥240 mg/dl or a history of taking lipid lowering medications. Smoking was dichotomized as ever or never smoking, physical activity as any leisure-time activity or none, and alcohol drinking as light to moderate (≥1 drink/month but <2 drinks/day), none or other [18]. Body mass index (BMI, kg/m^2^) and waist-to-hip ratio (WHR) were calculated based on the measured weight, height, waist circumference and hip circumference.

All subjects provided informed consent to participate, and the study was approved by the Institutional Review Boards of Columbia University in New York and the University of Miami.

### Carotid Plaque Ultrasound Imaging

High-definition carotid ultrasounography was performed according to a standard scanning and reading protocol by a sonologist trained and certified in performing ultrasound research studies. Detailed descriptions of the methods and reliability studies have been published previously [16]. In brief, carotid ultrasound imaging was performed on a GE LogIQ 700 system with a multifrequency 9/13-MHz linear-array transducer. Both internal and common carotid arteries and the bifurcations were examined for the presence of atherosclerotic plaque, defined as an area of focal wall thickening more than 50% greater than surrounding wall thickness. The sum of plaques insonated in all carotid artery segments was also analyzed.

### Genotyping

We analyzed variants of the SIRT and UCP genes available from a genome-wide association study performed on the Genome-Wide Human SNP Array 6.0 chip (AffyMetrix). DNA samples were processed according to Affymetrix procedures. The arrays were scanned on the GeneChip Scanner 3000 7G. Image data were analyzed using the Genotyping Console™. Vigorous quality control was applied to the samples and SNPs. Samples were removed from further analysis if they had call rates below 95%, relatedness, sex discrepancies, or were outliers beyond 6 SD from the mean using EIGENSTRAT [19]. SNPs with severe deviation from Hardy-Weinberg equilibrium (p<1E-06) or a genotyping call rate less than 95% were also removed using PLINK 1.05 [20]. After quality control, a total of 85 UCP/SIRT SNPs in the 11 UCP and SIRT genes were included in the final analysis of this study (table S1).

### Statistical Analyses

To reduce potential bias due to population stratification, we first performed principal component analysis to examine population substructure using EIGENSTRAT and selected the plaque associated principle components (PCAs) as genomic control variables. Univariate analysis was performed to identify demographic characteristics and RF associated with presence of carotid plaque (p<0.05) in order to include significant factors as covariates in the final genetic association analysis of the UCP and SIRT variants.

For single SNP-based association analyses, we examined the additive genetic effects of the UCP and SIRT variants on the presence of carotid plaque using logistic regression models and on the number of carotid plaques using Poisson regression models. These models were adjusted for age, sex, smoking, hypertension, diabetes and WHR, as well as for genomic control variable PCA2 and PCA4. PCAs associated with carotid plaque among the top 5 PCAs (p<0.05 for PCA2 and PCA4, and p>0.05 for PCA1, PCA3, PCA5). Zero-inflation modelling did not detect an excess of zeros in the distribution of plaque numbers in Poisson regression analysis.

For haplotype-based analysis, linkage disequilibrium (LD) blocks were first identified for each gene using Haploview [21]. Haplotypes for the SNPs in the LD block were then estimated for each subject using the E-M algorithm in PLINK. Similar to SNP-based analysis, an additive effect of a haplotype was examined using generalized linear regression models via SAS GENMOD procedure by coding 0, 1 and 2 based on the copy number of the haplotype. We limited haplotype analysis to the genes with a SNP showing an association after correction for multiple testing and to haplotypes with a frequency of at least 5%. All the analyses were performed using the generalized linear regression analysis procedure GENMOD in SAS 9.2 (SAS Institute Inc., Cary, NC, USA).

Bonferroni correction was employed to control for multiple testing based on the effective number of tests. For SNP-based analysis, the effective number of tests was defined as the sum of the LD blocks and singleton SNPs [22]. A total of 16 LD blocks were identified and 14 SNPs were not located in any LD block (Figure S1), leading to an effective test number of 30. For haplotype-based analysis, the analysis was limited to the genes with a SNP showing an association after correction for multiple testing to the haplotypes with a frequency of at least 5%. Therefore, a total of 23 haplotypes were investigated.

To explore sex and RF effect modification on the associations between gene variants and plaque, we examined SNP-by-sex and SNP-by-RF interactions and performed stratified analyses if the interaction terms in the models had p≤0.01. We also explored SNP-by-SNP interactions between the SNPs significantly associated with plaque and the SNPs in other genes and then conducted stratified analysis if the p value for interaction term was ≤0.01.

## Results

### Subject Characteristics, Vascular Risk Factors and Carotid Plaque Presence

**Figure 1:**
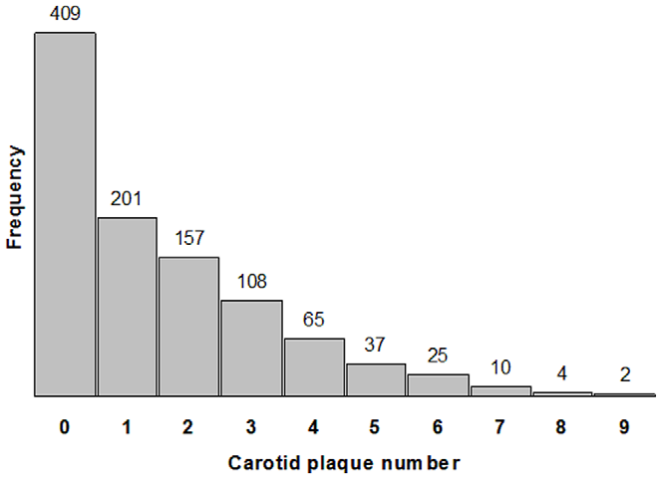
Distribution of carotid plaque number. Plot shows the frequency of subjects against their presence of carotid plaque number over 12 carotid artery segments analyzed.

Among 1018 subjects (mean age: 70±9), 61% were women, 67% Caribbean Hispanic, 17% Black, and 15% White. Overall, 60% of subjects had carotid plaques, 25% had three or more plaques (Figure 1), 62% had hypertension, 18% had diabetes, and 52% were smokers. Univariate analysis showed that age, sex, race/ethnicity, smoking, hypertension, diabetes, and waist-to-hip ratio were associated with the presence of carotid plaque (Table 1).

**Table 1:** Demographics and clinical characteristics of the study population.

		Carotid plaque		
Characteristics	All (n = 1018)	Absence (n = 409)	Presence (n = 609)	p
Age (yrs), Mean ± SD	69.71±8.84	66.34±8.13	71.98±8.58	<0.0001
Female, %	60.71	64.79	57.96	0.03
Race/ethnicity, %				0.002
White	14.83	10.76	17.57	
Black	16.6	14.67	17.9	
Hispanics	66.8	72.62	62.89	
Other	1.77	1.96	1.64	
Education attainment (yrs), %				0.4
≤8	43.91	46.45	42.2	
12-Sep	26.42	25.43	27.09	
>12	29.67	28.12	30.71	
Ever smoked, %	52.16	43.28	58.13	<.0001
Moderate alcohol drinking, %	41.36	41.32	41.38	0.98
No leisure-time physical activity, %	43.71	42.54	44.5	0.88
Hypertension (yes), %	62.28	55.26	67	0.0002
Diabetes (yes), %	18.47	14.18	21.35	0.004
Hypercholesterolemia (yes), %	27.11	24.21	29.06	0.09
BMI (kg/m^2^), Mean ± SD	28.56±5.03	28.76±5.02	28.43±5.04	0.31
WHR, Mean ± SD	0.92±0.08	0.91±0.08	0.93±0.08	0.0008

### Associations of SNPs with Presence of Carotid Plaque and with a Number of Carotid Plaque

The SNPs significantly associated with the presence and number of carotid plaques (p≤0.05 adjusted for multiple testing) are reported in Table 2. Two SNPs, rs107251 in SIRT6 and rs5977238 in UCP5, were associated with both presence and number of carotid plaque after correction for multiple testing. T-carriers of SIRT6 SNP rs107251 had an increased risk (OR 1.71, 95% CI = 1.23–2.37, adjusted p = 0.03) for carotid plaque presence and for a number of carotid plaques (RR 1.31, 95% CI = 1.18–1.45, adjusted p = 1.4×10^−5^); whereas T-carriers of UCP5 SNP rs5977238 had a decreased risk (OR 0.49, 95% CI = 0.32–0.74, adjusted p = 0.02) for carotid plaque presence and for a number of plaques (RR 0.64, 95% CI = 0.52–0.78, adjusted p = 4.9×10^−4^). Two additional SNPs (rs4712032 and rs12216101) in the SIRT5 gene were associated with a greater number of carotid plaques after correction for multiple testing.

**Table 2:** SNPs associated with presence or number of carotid plaque (Bonferroni-corrected p≤0.05).

Carotid plaque	Chr	Gene	SNP	Position (Mb)	Location	Minor allele (Freq.)	exp(beta) (95% CI)*	Nominal p	Adjusted p∧
Presence	19	*SIRT6*	rs107251	4127085	Intron	T (0.11)	1.71 (1.23–2.37)	0.001	0.03
	X	*UCP5*	rs5977238	129308417	Intron	T (0.07)	0.49 (0.32–0.74)	0.0008	0.02
Number	6	*SIRT5*	rs4712032	13662022	Flanking	G (0.31)	1.14 (1.06–1.22)	0.0007	0.002
	6	*SIRT5*	rs12216101	13669021	Flanking	G (0.21)	1.16 (1.07–1.26)	0.0004	0.01
	19	*SIRT6*	rs107251	4127085	Intron	T (0.11)	1.31 (1.18–1.45)	4.8×10^−7^	1.4×10^−5^
	X	*UCP5*	rs5977238	129308417	Intron	T (0.07)	0.64 (0.52–0.78)	1.6×10^−5^	4.9×10^−4^

*Beta was estimated based on Logistic and Poisson regression, respectively, for presence and number of carotid plaque after adjusting for age, sex, smoking, hypertension, diabetes and waist-to-hip ratio as well as genomic control variables (PCAs).

∧ Bonferroni-corrected p was based on the sum of singleton SNPs and LD blocks.

### Haplotypes Associated with the Presence or Number of Carotid Plaque

The results of the haplotypes analyses are presented in Table 3. Two haplotypes, TT at rs107251 and rs3760905 in the SIRT6 gene and TTTCACATT at 9 SNPs in the UCP5 gene (Figure S1), were associated with both carotid plaque phenotypes. Haplotypes within the SIRT6 gene were associated with increased risk of carotid phenotypes while within the UCP5 gene were protective. Also, a 4-SNP haplotype GCGG of the SIRT5 gene was associated with a greater number of carotid plaques (RR 1.16, 95% CI = 1.07–1.26, adjusted p = 0.009).

**Table 3:** Haplotypes in the SIRT5, SIRT6 and UCP5 genes associated with carotid plaque (Bonferroni-corrected p≤0.05).

Gene	SNPs	Haplotype	Freq.	Carotid plaque	exp(beta) (95% CI)*	Nominal p	Adjusted p∧
*SIRT5*	rs4712032, rs12175268, rs10498683, rs12216101 (LD block 3)	GCGG	0.21	Number	1.16 (1.07–1.26)	0.0004	0.009
*SIRT6*	rs107251, rs3760905 (LD block 13)	TT	0.11	Presence	1.67 (1.20–2.32)	0.002	0.05
				Number	1.31 (1.18–1.45)	5.0×10^−7^	1.1×10^−5^
*UCP5*	rs6418932, rs12557276, rs2235800, rs16999665, rs5977248, rs4830187, rs4829716, rs4830188, rs5932754 (LD Block 16)	TTTCACATT	0.07	Presence	0.45 (0.30–0.68)	0.0002	0.005
				Number	0.65 (0.64–0.78)	6.4×10^−6^	1.5×10^−4^

*beta was estimated based on Logistic and Poisson regression, respectively, for presence and number of carotid plaque after adjusting for age, sex, smoking, hypertension, diabetes and waist-to-hip ratio as well as genomic control variables (PCAs).

∧ Bonferroni corrected p based on 23 tested haplotypes with a frequency>5% in SIRT5, SIR6 and UCP5 genes.

### Interactions between the SIRT/UCP gene Variants and Vascular Risk Factors

Table 4 shows the interactions between SNPs and vascular risk factors with a nominal p≤0.01 and the genetic effects stratified by the status of the specific vascular risk factor.

**Table 4:** SNPs showing interaction with sex and vascular risk factors for presence or number of carotid plaque with a nominal p≤0.01.

			Interaction analysis*				Stratified analysis exp(beta) (95% CI) for 1 minor allele change	
Carotid plaque	Vascular risk factor (VRF)	SNP (gene)	P_vrf_	P_snp_	P_interaction_	exp(beta) for interaction (95% CI)	With VRF	Without VRF
Presence	Female	rs1685356 (UCP3)	0.01	0.02	0.007	1.81 (1.17–2.79)	1.42 (1.08–1.87)	0.76 (0.54–1.08)
		rs1726745 (UCP3)	0.01	0.03	0.01	1.71 (1.13–2.59)	1.38 (1.07–1.77)	0.79 (0.56–1.10)
Number	Female	rs1430579 (UCP1)	0.0004	0.0001	0.0003	1.30 (1.13–1.50)	1.06 (0.97–1.16)	0.80 (0.72–0.90)
		rs1472268 (UCP1)	0.0006	0.0002	0.0005	1.29 (1.12–1.49)	1.05 (0.96–1.15)	0.80 (0.72–0.90)
		rs1472269 (UCP1)	0.0005	<.0001	0.0005	1.30 (1.12–1.51)	1.02 (0.93–1.13)	0.78 (0.69–0.88)
		rs6829571 (UCP1)	0.0005	0.0003	0.0006	1.29 (1.12–1.49)	1.07 (0.97–1.17)	0.82 (0.73–0.91)
	Smoking	rs2841503 (SIRT5)	<.0001	0.0003	0.003	0.67 (0.52–0.87)	0.98 (0.84–1.15)	1.47 (1.20–1.82)
		rs6907892 (UCP4)	<.0001	0.05	0.005	0.80 (0.69–0.93)	0.91 (0.82–0.99)	1.12 (1.00–1.26)
	Diabetes	rs10498683 (SIRT5)	<.0001	0.94	0.003	0.67 (0.51–0.87)	0.71 (0.55–0.90)	1.00 (0.89–1.12)
		rs6418932 (UCP5)	<.0001	0.28	0.006	0.77 (0.65–0.93)	0.84 (0.71–1.00)	1.04 (0.95–1.14)
		rs12557276 (UCP5)	<.0001	0.002	0.008	0.76 (0.61–0.93)	0.90 (0.74–1.10)	1.14 (1.04–1.25)
		rs2235800 (UCP5)	<.0001	0.25	0.004	0.76 (0.63–0.92)	0.82 (0.69–0.98)	1.05 (0.95–1.15)
		rs4830187 (UCP5)	<.0001	0.002	0.004	0.74 (0.60–0.91)	0.88 (0.72–1.07)	1.14 (1.04–1.25)
		rs4830188 (UCP5)	<.0001	0.34	0.006	0.76 (0.62–0.92)	0.80 (0.66–0.97)	1.04 (0.94–1.14)
		rs5932754 (UCP5)	<.0001	0.37	0.009	0.77 (0.63–0.94)	0.82 (0.68–0.99)	1.03 (0.94–1.13)
	Hypertension	rs10498683 (SIRT5)	<.0001	0.12	0.005	0.73 (0.58–0.91)	0.84 (0.74–0.95)	1.15 (0.96–1.38)
		rs9370232 (SIRT5)	<.0001	0.21	0.0004	0.69 (0.56–0.85)	0.77 (0.69–0.87)	1.12 (0.94–1.33)
		rs536715 (SIRT3)	0.003	0.0006	0.01	1.39 (1.08–1.78)	0.95 (0.85–1.07)	0.67 (0.54–0.84)
		rs5977238 (UCP5)	<.0001	1	0.001	0.51 (0.34–0.77)	0.51 (0.39–0.67)	0.95 (0.69–1.30)

*P_vrf_, P_snp_, P_interaction_ and beta were, respectively, p-value for risk factor main effect, p-value for SNP main effect, p-value and regression coefficient for their interactive effect, based on Logistic (plaque presence) and Poisson (plaque count) regression models after adjusting for age, sex, smoking, hypertension, diabetes and waist-to-hip ratio as well as genomic control variables (PCAs) if applicable.

For plaque presence, SNP-by-sex interaction was found for 2 potential regulatory UCP3 SNPs (rs1685356 and rs1726745; p≤0.01). Specifically, increased risk was found for A-carrier women at UCP3 SNP rs1685356 (OR 1.42, 95%CI 1.08–1.87) but not for A-carrier men (OR 0.76, 95%CI 0.54–1.08), and decreased risk was found for C-carrier men at UCP1 SNP rs1430579 (OR 0.80, 95%CI 0.72–0.90) but not for women (RR 1.06, 95%CI 0.97–1.16). No interaction with RF was found with a nominal p≤0.01.

For plaque number, a SNP-by-sex interaction was found for 4 UCP1 SNPs (rs1430579, rs1472268, rs1472269, and rs6829571; p≤0.0006) at transcription factor binding sites (TFBS) (http://snpinfo.niehs.nih.gov/snpfunc.htm). The associations of several SNPs in UCP4, 5, and SIRT3, 5 with plaque number varied by smoking status, diabetes and hypertension. For example, minor allele carriers at SIRT5 SNP rs2841503 had an increased risk for great number of plaques among non-smokers (RR 1.47, 95%CI 1.20–1.82). Likewise, minor allele carriers at several UCP5 SNPs had a decreased risk of plaque number in those with diabetes but increased risk in individuals without diabetes.

### Interactions between Genetic Variants

Table 5 shows the interactions with a nominal p≤0.01 of the two most significant SNPs (rs107251 in SIRT6 and rs5977238 in UCP5) with the SNPs in the other UCP and SIRT genes. Among T-carriers of UCP5 rs5977238, individuals with the minor allele at three UCP1, 2 SIRT3 and 1 SIRT5 SNPs had an increased risk of having plaque. Among T-carriers at SIRT6 rs107251, individuals with minor allele at two UCP5 SNPs had a lower risk of having plaque.

**Table 5:** SNP×SNP interactions for presence and number of carotid plaque with a nominal p≤0.01.

			Interaction analysis*				Stratified analysis exp(beta) (95% CI) for 1 minor allele change at SNP2	
Carotid plaque	SNP1 (gene)	SNP2 (gene)	P_snp1_	P_snp2_	P_interaction_	exp(beta) for interaction (95% CI)	With minor allele at SNP1	Without minor allele at SNP1
Presence	rs5977238	rs1430579 (UCP1)	<.0001	0.05	0.0007	2.64 (1.51–4.60)	2.62 (1.29–5.33)	0.81 (0.66–1.00)
	(UCP5)	rs1472268 (UCP1)	<.0001	0.04	0.0007	2.59 (1.49–4.51)	2.55 (1.26–5.18)	0.81 (0.66–1.00)
		rs1472269 (UCP1)	<.0001	0.02	0.002	2.47 (1.40–4.33)	2.40 (1.17–4.90)	0.78 (0.63–0.96)
		rs6829571 (UCP1)	<.0001	0.09	0.002	2.46 (1.41–4.31)	2.43 (1.18–5.01)	0.84 (0.68–1.04)
Number	rs107251	rs7065731 (UCP5)	<.0001	0.79	0.006	0.35 (0.16–0.74)	0.34 (0.16–0.73)	1.03 (0.89–1.21)
	(SIRT6)	rs16999665 (UCP5)	<.0001	0.94	0.01	0.27 (0.10–0.73)	0.26 (0.10–0.70)	1.00 (0.84–1.20)
	rs5977238	rs1430579 (UCP1)	<.0001	0.11	0.006	1.43 (1.11–1.83)	1.62 (1.14–2.30)	0.94 (0.87–1.01)
	(UCP5)	rs1472268 (UCP1)	<.0001	0.09	0.006	1.42 (1.11–1.83)	1.61 (1.14–2.28)	0.93 (0.87–1.01)
		rs1472269 (UCP1)	<.0001	0.02	0.008	1.41 (1.09–1.83)	1.60 (1.13–2.29)	0.91 (0.84–0.98)
		rs16874223 (SIRT5)	<.0001	0.78	0.001	1.55 (1.19–2.01)	1.52 (0.91–2.55)	0.98 (0.86–1.12)
		rs11596401 (SIRT1)	0.03	0.92	0.004	0.60 (0.42–0.85)	0.55 (0.36–0.86)	1.00 (0.93–1.07)
		rs11246007 (SIRT3)	<.0001	0.36	0.005	1.48 (1.13–1.94)	1.72 (1.17–2.52)	0.95 (0.86–1.06)
		rs1023430 (SIRT3)	<.0001	0.66	0.0008	1.43 (1.16–1.77)	1.67 (1.19–2.35)	0.98 (0.89–1.08)

*P_snp1_, P_snp2_, P_interaction_ and beta were, respectively, p-value for SNP1 main effect, p-value for SNP2 main effect, p-value and regression coefficient for their interactive effect, based on Logistic (plaque presence) and Poisson (plaque count) regression models after adjusting for age, sex, smoking, hypertension, diabetes and waist-to-hip ratio as well as genomic control variables (PCAs) if applicable.

## Discussion

In this study we report on the association of the UCP5, SIRT6 and SIRT5 gene variants with carotid plaque, a surrogate marker of atherosclerosis. Haplotype analyses confirmed and straightened these observations. Several important effect modifications of these relationships were found by sex (for the associations with UCP1 and UCP3) and RF including smoking (for the associations with SIRT5 and UCP4), hypertension (for the associations with SIRT3, SIRT5, and UCP5), and diabetes (for the associations with SIRT5 and UCP5). Some gene-gene interactions have also shown among UCP5 and genetic variants in UCP1, SIRT1, SIRT3, and SIRT5 resulting in an increased risk of having plaque; and among SIRT6 and genetic variants in UCP5 leading to a decreased risk of having plaque. These results suggest that genetic variants in sirtuins and UCP genes may have an influence on the development of vascular aging phenotypes, independent of common RF.

The proteins expressed by the SIRT and UCP genes have been involved in the mechanisms leading to aging and age-dependent atherosclerosis [6], [23]. Age-dependent arterial wall phenotypic changes make cardiovascular system more susceptible to oxidative damage and an increased risk of CVD even in the absence of traditional RF. Several mechanisms underlay vascular aging [5]. Oxidative stress is one of the main mechanisms leading to overt atherosclerosis in elderly [24]. Age-dependent mitochondrial impairment, especially in the function of proteins that regulates the mitochondrial physiology, such as UCPs, is fundamental for ROS mediated cell damage [25]. Sirtuins are histone deacetylases that are implicated in many cellular processes including cell cycle regulation, fatty acid metabolism, lifespan regulation and apoptosis [6]. There is growing evidence that UCPs and SIRTs may be involved in the mechanisms leading to atherosclerosis by ROS production with aging [6], [23], [26].

One of our main findings is that the variants in SIRT5 and SIRT6 genes were significantly associated with the risk of carotid plaque. SIRT5 is a mitochondrial sirtuin that is upregulated by caloric restriction and is involved in mitochondrial ROS production regulation [27]. The mains substrates of SIRT5, cytochrome c and carbamoyl phosphate synthase suggest that SIRT5 may have role in controlling the atherosclerotic process [28], [29]. SIRT6 is predominantly localized in the cellular nucleus and it is highly expressed in heart and brain [30]. Similar to SIRT5, SIRT6 is involved in DNA repair and lifespan extension although the exact mechanism is not fully elucidated [31]. The emerging role of SIRT6 in promoting proper chromatin function in several physiologic contexts, including telomere and genome stabilization, is DNA repair pathway involved in repairing ROS-induced DNA damage [32], as well as in preventing aging phenotypes [33]. SIRT6 is also a key regulator of glucose homeostasis [34]. Recently, it has been showed that mice deficient in SIRT6 develop abnormalities usually associated with premature aging phenotypes, including several metabolic defects, such as increase in fat accumulation, impaired glucose tolerance, and alteration in lipid homeostasis [35]. The main targets of SIRT6 are nuclear factor-kappa B (NF-κB), a transcription factor that plays pivotal roles in regulating aging, and inflammation as well as hypoxia-inducible transcription factor HIF1α, an important regulator of glucose homeostasis that has been also linked to lifespan regulation [32]. The SIRT6 polymorphism identified in our study may well be interfering with NF-κB or HIF1α and contribute this way to an accelerated vascular aging.

Another main finding is the significant association between UCP5 variant rs5977238 and the risk of carotid plaque. UCPs have a pivotal role in ischemia and atherosclerosis by controlling ROS production [10], [36]. Although the physiological function of UCP5 has not yet been fully established, it causes mild uncoupling by which it may diminish mitochondrial superoxide production, hence protecting against oxidative damage [37]. By attenuating superoxide generation and maintaining oxidative phosphorylation UCP5 may be play a protective role against atherosclerosis and CVD. Interestingly, intergenic interaction analyses showed that among T-carriers of UCP5 rs5977238, individuals with the minor allele at the SNPs at the UCP1, SIRT3 and SIRT5 genes had an increased risk of having plaque. Sirtuins may control the genetic expression of UCPs by binding directly to their promoter [38], [39]. Relationships between SIRTs and UCPs have been also demonstrated in delay age-related disease by activating cellular metabolism [40]. A recent study has indicated that UCP5 controls the cellular mitochondrial membrane potential, ATP production and oxygen consumption [37]. This may interact with the SIRT3 and SIRT5, which are mitochondrial SIRTs, in controlling ROS production and therefore the development of atherosclerosis. SIRT3 expression significantly increases in response to oxidative stress and protects again ROS-mediated damage [41]. UCP1 expression increases superoxide production and decreases the availability of NO, evidence of oxidative stress, resulting in inefficient blood vessel metabolism that can cause vascular aging [9]. The synergistic effect of SIRTs/UCPs might be necessary to maintain oxidative balance and ATP production, and consequently cellular survival and modification in response to different stimuli, such as oxidative stress.

In the present study, we observed sex-specific effects of genetic variants in UCP1 and 3 genes on presence of carotid plaque in women. Difference in UCP3 exon 5 variants have been observed between African and white American women and suggested a role of UCP3 for the higher predisposition of obesity in African American women [42]. Interestingly, an experimental study has demonstrated an increase of UCP1 expression in female rats under stress [43]. Polymorphisms of UCP1 have been associated with fat metabolism, obesity, and diabetes [44], [45], which are known to have different distributions in men and women. Sex hormones have been also shown to modulate UCP1 and UCP3 expression [46]. These findings may in part explain the sex-specific effect of UCP1 and 3 genetic variants on carotid plaque.

Smoking is one of the major lifestyle vascular factors. Life-long cigarette smokers have a higher prevalence of atherosclerosis and CVD through the increase of oxidative stress [47]. We found an interaction between smoking and SIRT5 and UCP4 variants for a decreased risk of plaque presence and plaque number. These interactions with smoking may be due to the control of ROS production. A direct association between smoking and UCPs has been found in mice where cigarette smoke exposure increased UCPs in the brown fat [48]. Further studies are needed to clarify the exact interaction between smoking and SIRTs and UCPs.

Some modifications by hypertension and diabetes were also observed in the association of SIRT3, SIRT5 and UCP5 genetic variants with carotid plaque. In experimental studies, SIRT3 expression has been regulated by Angiotensin II, which may play a pivotal role in the etiology of hypertension [49], [50]. UCPs have shown to lower blood pressure in obese mice, suggesting a potential protective effect of UCP on atherosclerosis in type 2 diabetes [51]. Moreover, as well as SIRTs, UCPs genetic modulation in hypertensive rats have been demonstrated to have a cardioprotective effect by interacting with Angiotensin II in controlling ROS production [52]. The role of SIRTs and UCPs in diabetes has also been previously explored [53], [54]. and linked to atherosclerosis due to their direct effect on insulin release and regulation of the glucose substrate consumption in the endothelial cells by reducing ROS production [55].

Our results need to be taken with caution because of several limitations. First, our study included a relative small convenience sample with a few available SNPs in the sirtuin and UCP genes (7 sirtuin and 5 UCP genes for a total of 3055 SNPs, http://www.ncbi.nlm.nih.gov/snp). Second, the multiethnic nature and the heterogeneous origin of the study population may reduce the statistical power even thought using PCAs as genomic control in the analyses. Third, the interaction analyses were not adjusted for multiple testing and highly exploratory. Fourth, some traditional risk factors such as smoking, hypercholesterolemia and physical activity were dichotomized representing crude measures of these exposures. Despite these limitations, we observed novel associations of genetic variants in UCP1, 3, 4, and 5 and SIRT3, 5, and 6 with carotid plaque. Exploring the impact of these genes on vascular aging and premature atherosclerosis may be of particular importance for detecting asymptomatic individuals at increased risk for vascular disease. Moreover, it may aid to the development of a novel vascular preventive compounds such as resveratrol, a SIRT activator [56] in order to restore the impaired molecular pathways, and thereby reduce atherosclerosis. Further research is imperative to confirm these findings.

## Supporting Information

Figure S1 **Linkage disequilibrium (LD) pattern in 10 sirtuin and mitochondrial uncoupling protein genes.** Haploview program is used to calculate the D′. Shown in each box are estimated statistics of the D′, which indicates the LD relationship between each pair of single nucleotide polymorphisms (SNPs) in each gene and are not labeled if D′ = 1.00.
(TIF)

Table S1 **SIRT/UCP localization and biological effects on cardiovascular disease.**
(DOC)
