# Supplementary figures and images for: Association of the Sirtuin and Mitochondrial Uncoupling Protein Genes with Carotid Plaque
Source: PLoS One. 2011 Nov 7;6(11):e27157. doi: 10.1371/journal.pone.0027157 (PMC3210138; doi:10.1371/journal.pone.0027157)

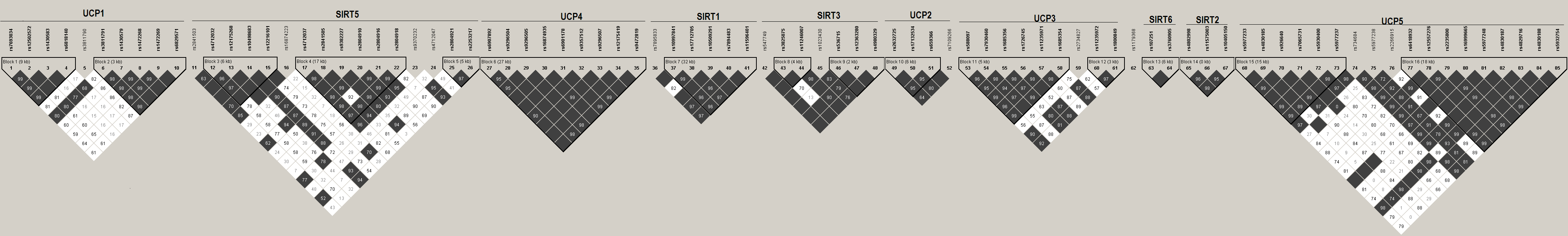

Supplement: Figure S1 — Linkage disequilibrium (LD) pattern in 10 sirtuin and mitochondrial uncoupling protein genes. Haploview program is used to calculate the D′. Shown in each box are estimated statistics of the D′, which indicates the LD relationship between each pair of single nucleotide polymorphisms (SNPs) in each gene and are not labeled if D′ = 1.00. (TIF) [file pone.0027157.s001.tif]
