# Supplementary material for: Association of the Sirtuin and Mitochondrial Uncoupling Protein Genes with Carotid Plaque
Source: PLoS One. 2011 Nov 7;6(11):e27157. doi: 10.1371/journal.pone.0027157 (PMC3210138; doi:10.1371/journal.pone.0027157)
Supplement: Table S1 — SIRT/UCP localization and biological effects on cardiovascular disease. (DOC) [file pone.0027157.s002.doc]

| **Table S1.** SIRT/UCP Localization and Biological Effects on Cardiovascular Disease (CVD) | | | | |
| --- | --- | --- | --- | --- |
| Gene | Position | Cellular Localization | Biological effects on CVD | No. of tested SNPs |
| *SIRT1* | 10q21 | Nucleus | Aging, Inflammation, Blood lipids, Insulin release and action, Obesity, Atherosclerosis, Endothelium damage | 6 |
| *SIRT2* | 19q13 | Cytoplasm | Lipids accumulation, Adipocyte differentiation | 3 |
| *SIRT3* | 11p15 | Mitochondria | Aging, Mitochondria energy homeostasis | 7 |
| *SIRT4* | 12q24 | Mitochondria | Insulin secretion | 1 |
| *SIRT5* | 6p23 | Mitochondria | Mitochondria energy homeostasis | 16 |
| *SIRT6* | 19p13 | Nucleus | Aging, Metabolism, DNA repair | 2 |
| *UCP1* | 4q31 | Mitochondria | Oxidative Stress, Fat metabolism, Diabetes, Obesity, Metabolic Syndrome | 10 |
| *UCP2* | 11q13 | Mitochondria | Oxidative Stress, Type2 diabetes, Obesity, HDL Cholesterol, Hyperglycemia, Inflammation, Smoking, Metabolic Syndrome, Atherosclerosis | 4 |
| *UCP3* | 11q13 | Mitochondria | Oxidative Stress, Type2 diabetes, Obesity, HDL Cholesterol, Hyperglycemia, Inflammation, Smoking, Metabolic Syndrome | 9 |
| *UCP4* | 6q12 | Mitochondria | Oxidative Stress, Apoptosis in neurons | 9 |
| *UCP5* | Xq24 | Mitochondria | Oxidative Stress, Neurodegeneration | 18 |
